# Trajectory of the systemic immune-inflammation index and in-hospital mortality in patients with sepsis

**DOI:** 10.3389/fcimb.2025.1616538

**Published:** 2025-09-23

**Authors:** Wanling Xu, Shurui Ren, Zheng Li, Li Pang

**Affiliations:** Department of Emergency Medicine, The First Hospital of Jilin University, Changchun, Jilin, China

**Keywords:** sepsis, systemic immune-inflammation index, restricted cubic spline, predictive model, in-hospital mortality

## Abstract

**Background:**

Sepsis is a complex systemic inflammatory response syndrome triggered by infection with high morbidity and mortality. The systemic immune-inflammation index (SII) is a biomarker of inflammation and immune status. This study investigated the relationship between the SII trajectory and in-hospital mortality in patients with sepsis.

**Methods:**

This retrospective study included 1015 adults who were admitted via the emergency department of the First Hospital of Jilin University with a first episode of sepsis between June 2018 and February 2025. Latent-class mixed models (LCMM) were used to identify SII trajectory subgroups, and Cox regression was used to analyze the relationship between subgroups and in-hospital mortality. An eXtreme Gradient Boosting (XGBoost) machine learning model was used to quantify the effect of each variable on the risk of in-hospital mortality. Restricted cubic spline (RCS) analysis assessed the nonlinear relationship between SII and in-hospital mortality.

**Results:**

LCMM analysis identified five SII trajectory subgroups. Cox regression analysis showed that Class 1 (the group with continuous increase in SII from a low to medium level), Class 3 (the group with a stable decline in SII from a high level), Class 4 (the group with a stable high SII level) and Class 5 (the group with a stable medium SII level) all had higher risk of in-hospital mortality than Class 2 (the group with a stable medium-high SII level). Class 1 and Class 4 had the highest risk of in-hospital mortality (hazard ratio [HR] 15.14 and 6.31, respectively). The XGBoost model confirmed that the SII trajectories were independent predictors of in-hospital mortality. The RCS analysis revealed a U-shaped relationship between the SII within 24 hours after admission and in-hospital mortality, with both low and high SII levels associated with higher in-hospital mortality.

**Conclusions:**

In patients with sepsis, the risk of in-hospital mortality differs according to the SII within 24 hours of admission and the SII trajectory. The risk of in-hospital mortality was greatest in patients whose SII increased continuously and those whose SII stabilized at a high level, and was lowest in patients with an SII stabilized at a medium-high level. The SII within 24 hours after admission had a U-shaped relationship with in-hospital mortality.

## 1 Introduction

Sepsis is a complex systemic inflammatory response syndrome triggered by infection, characterized by complex pathophysiological processes, including immune imbalances, endothelial damage, and metabolic disorders (Evans et al., 2021).Sepsis is one of the most common life-threatening diseases among patients admitted to emergency departments and intensive care units (ICUs), with a mortality rate of up to 26%, and is associated with high medical costs (Rudd et al., 2020).The Third International Consensus Definition of Sepsis (Sepsis 3.0) guidelines emphasize the importance of early risk screening and prognosis assessment in patients with sepsis (Singer et al., 2016).

The systemic immune-inflammatory index (SII) is an inflammatory indicator calculated based on the lymphocyte, neutrophil, and platelet counts. As a derivative indicator based on routine blood tests, SII has the advantages of cost-effectiveness and clinical availability, and reflects both the inflammation and immune status of patients (Jiang et al., 2023). SII plays an important role in the diagnosis and treatment of a variety of diseases, and in cardiovascular disease SII can predict the occurrence of adverse cardiovascular events (Tang et al., 2021). In addition, SII is significantly associated with the occurrence of rheumatoid arthritis and other rheumatological diseases (Liu B. et al., 2023; Ma et al., 2024), and chronic kidney disease prevalence and mortality. In the field of oncology, SII is an independent predictor of treatment response and survival (Kou et al., 2023). SII is also a valuable indicator in the field of sepsis. SII combined with the Systemic Organ Failure Assessment (SOFA) or quick SOFA (qSOFA) score, or SII combined with procalcitonin are superior to any single indicator for predicting the prognosis in patients with sepsis (Liu C. et al., 2023; Mangalesh et al., 2023; Li et al., 2024). Moreover, in patients with sepsis, SII is an independent risk factor for in-hospital mortality, with both low and high SII levels being associated with an increased risk of in-hospital mortality (Liu C. et al., 2023).

However, most previous studies used SII data measured at a single time-point, without considering the dynamic nature of sepsis (Rabb et al., 2016), which is characterized by excessive inflammation in the early stage and changes in immunosuppression in the later stage (Bernsmeier et al., 2020; Liu et al., 2022). Failure to consider changes in the SII level may lead to the loss of key pathophysiological information. In contrast, latent-class mixed models (LCMM) are statistical models that analyze longitudinal data to reveal heterogeneity in the data by introducing latent classes and constructing mixed-effect models for each class separately to map out the unique trajectory pattern for each subgroup (Herle et al., 2020). This approach enables differences in disease progression to be identified in different subgroups based on differences in their trajectories.

Therefore, in order to better understand the dynamic trajectory of SII in patients with sepsis and its association with adverse outcomes, we conducted a retrospective cohort study to identify the relationship between SII trajectory and in-hospital mortality in patients with sepsis, and provide a scientific basis for personalized treatment.

## 2 Materials and methods

### 2.1 Study population

This study included adult patients with a first episode of sepsis, who were hospitalized in the emergency department of the First Hospital of Jilin University between June 2018 and February 2025. Sepsis was defined according to the Sepsis-3 criteria as follows (Singer et al., 2016): (i) confirmed or suspected infection; (ii) an increase of ≥ 2 points in the baseline SOFA score. The exclusion criteria included: (i) duration of hospitalization <48 hours; or (ii) <3 days of complete blood count data within the first 5 days after admission. The Medical Ethics Committee of the First Hospital of Jilin University approved this study (approval no. 2023-723), and we conducted the study following the ethical standards of the Declaration of Helsinki. The requirement for informed consent was waived owing to the retrospective study design.

### 2.2 Data collection and outcomes

We collected detailed data on the demographic and clinical characteristics of patients, including age, sex, comorbidities (including hypertension, diabetes, coronary atherosclerotic heart disease, chronic obstructive pulmonary disease, cerebrovascular disease, hematological diseases, malignancies, liver diseases [including cirrhosis, primary or secondary liver cancer], kidney diseases, and immune system diseases). In addition, data were collected on patients’ most abnormal complete blood counts (including white blood cell count, neutrophil count, lymphocyte count, platelet count, hemoglobin); C-reactive protein, prolactin, fibrinogen and alanine aminotransferase levels; activated partial prothrombin time, prothrombin time, International Normalized Ratio, within 24 hours of admission. Data were also collected on patients’ aspartate aminotransferase, lactic acid, albumin, total bilirubin, direct bilirubin, creatinine (Cr), potassium, sodium, chloride, calcium, N-terminal prohormone of brain natriuretic peptide, and troponin I levels; pH in blood gas analysis, oxygen partial pressure, carbon dioxide partial pressure, Sequential Organ Failure Assessment (SOFA) score, and whole blood cell counts for 5 consecutive days after admission. The primary outcome of this study was in-hospital mortality, and secondary outcomes were the need for mechanical ventilation and continuous renal replacement therapy, length of ICU stay, and total length of hospital stay.

### 2.3 Data processing

#### 2.3.1 Missing value processing

We excluded variables with a proportion of missing values greater than 20% (namely, prolactin, lactic acid, N-terminal prohormone of brain natriuretic peptide levels; pH in blood gas analysis, partial pressure of oxygen, and partial pressure of carbon dioxide). For the other missing data, we used the Multivariate Imputation by Chained Equations method, a predictive mean-matching technique based on the Multivariate Imputation by Chained Equations function in R language using five different interpolation modalities to impute the missing data.

#### 2.3.2 SII calculation

The SII was calculated daily for 5 consecutive days using the formula:


SII = P × N/L

where P, N and L are the platelet, neutrophil, and lymphocyte counts, respectively. As the original SII values were large, we used the logarithm-transformed values (Ln-SII) in the analysis to ensure statistical stability.

### 2.4 Statistical analysis

#### 2.4.1 Latent-class mixed model

All statistical analyses were performed using R software (v4.2.3). We used LCMM to analyze and classify the trajectory of SII changes in the patients with sepsis (Eriksson et al., 2021). LCMM is a robust statistical method designed to identify subgroups of patients with similar longitudinal trajectories. Using linear mixed models, the method captures trends at the individual level through random effects and uses latent category analysis to group patients according to common trajectories of change, effectively identifying patient subgroups with different characteristics. To select the optimal number of potential categories, we built LCMM models with 2 to 6 different categories in the cohort. We used the Akaike Information criteria, Bayesian information criteria (BIC), sample-adjusted BIC (SABIC), and entropy to evaluate the goodness-of-fit of the LCMM. The higher the entropy and the lower the Akaike Information criteria, BIC and SABIC, the better the model fit. We used entropy ≥0.80 as a selection criterion. In addition, in order to ensure the stability of the model, we set the sample size of each category to be >0.1% of the total study population, and confirmed the goodness-of-fit of the model by ensuring an average posterior probability of ≥70% for all categories.

#### 2.4.2 Comparison of the clinical features and results between subgroups

Continuous variables with a normal distribution were reported as the mean ± standard deviation, and the statistical significance of differences between subgroups was tested using analysis of variance. Continuous variables with a non-normal distribution were reported as the median and interquartile range (IQR), and the Kruskal-Wallis test was used to assess the statistical significance of differences between groups. Categorical variables were reported as frequencies and percentages, and the Chi-square test or Fisher’s exact test was used to assess the statistical significance of differences between groups. Two-tailed *P* values <0.05 were considered statistically significant.

#### 2.4.3 Survival analysis and machine learning model to evaluate the effect of the SII trajectory on in-hospital mortality in patients with sepsis

Kaplan-Meier survival curves were plotted to show the survival of subgroups with different SII trajectories, and the log-rank test was used to assess the significance of differences in survival between subgroups. In addition, an unadjusted Cox regression model was used to calculate the hazard ratio (HR) for in-hospital mortality among SII trajectory subgroups. To investigate the independent predictive value of SII trajectory subgroups for in-hospital mortality in patients with sepsis, forward stepwise Cox regression was used to screen variables. Age, kidney disease, platelet count, PT, International Normalized Ratio, and fibrinogen, were included in the multivariable Cox regression model. Subsequently, septic shock was forcibly incorporated into the model, and the multivariate Cox regression was repeated.

We used the eXtreme Gradient Boosting (XGBoost) machine-learning algorithm to quantify the effect of each risk factors on in-hospital mortality. The contribution of each variable to an individual’s predicted outcome was quantified using the Shapley additive explanation (SHAP) value, which visually depicted the relative importance of each variable to the risk of in-hospital mortality. In addition, we evaluated the sensitivity and specificity of the XGBoost model using receiver operating characteristic curve analysis, and the area under the curve was used to confirm the predictive value of the model.

#### 2.4.4 Restricted cubic spline analysis to characterize the nonlinear relationship between SII and in-hospital mortality in patients with sepsis

RCS plots were used to explore the potential nonlinear relationship between the mean SII within 24 hours of admission and the mean SII within 5 days of admission and in-hospital mortality in patients with sepsis. The corresponding HR and 95% confidence interval (CI) were calculated, and an RCS with four nodes was used to capture the nonlinear effect of SII on mortality, with the selection of nodes determined based on the criteria recommended in previous studies (Elhakeem et al., 2022) or by maximizing the goodness-of-fit of the model.

## 3 Results

### 3.1 SII trajectories and baseline features

**Figure 1:**
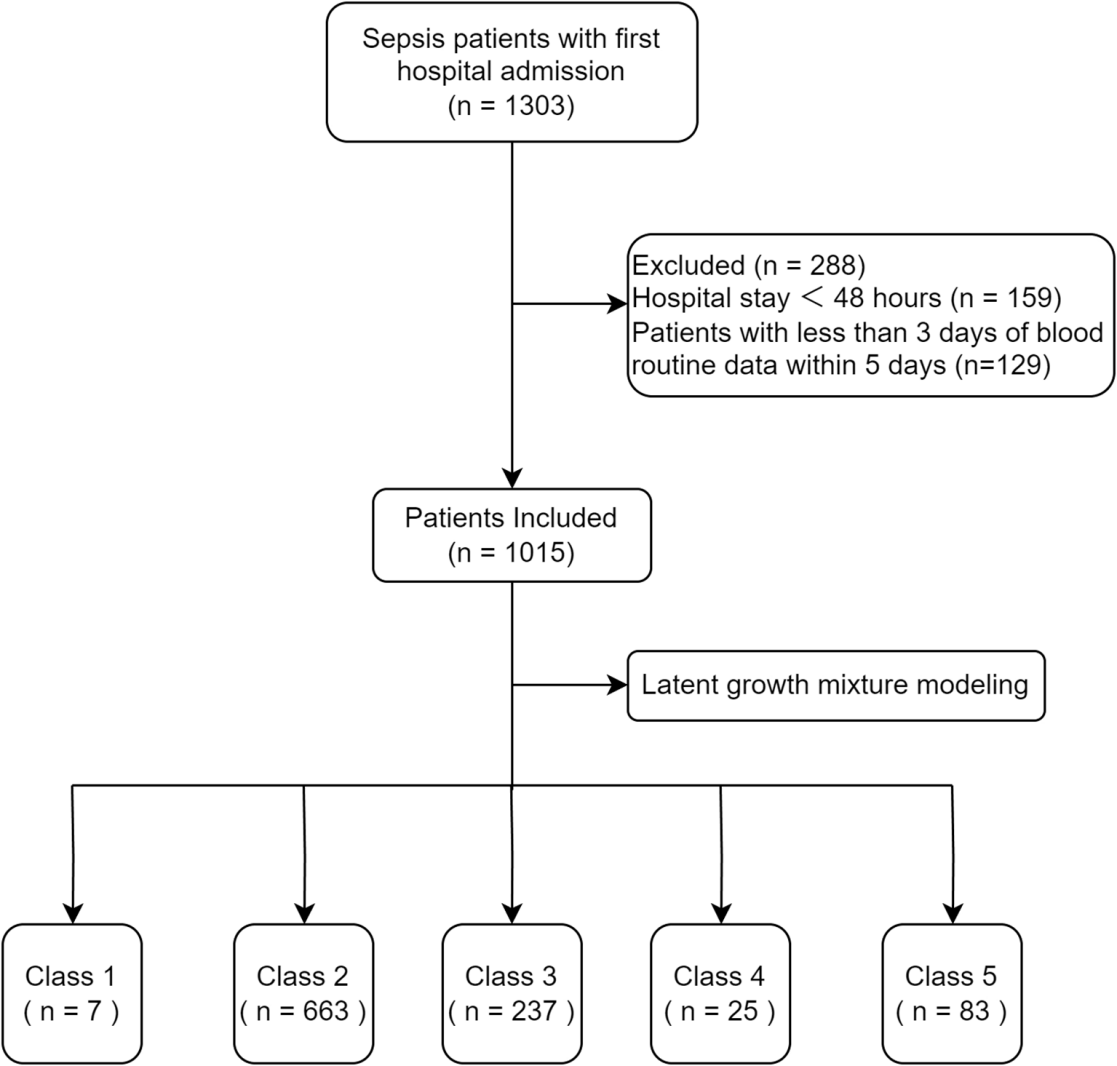
Flow chart of the study.

A total of 1015 patients were included in the analysis (**Figure 1**). The goodness-of-fit of the LCMMs are shown in **Supplementary Table S1**. The SABIC values decreased and the entropy increased from Model 1 to Model 6. The entropy of Model 5 and Model 6 was >0.8, but the average posterior probability of Class 2, Class 3, Class 4, Class 5, and Class 6 in Model 6 was <70%; therefore, we chose Model 5.

**Figure 2:**
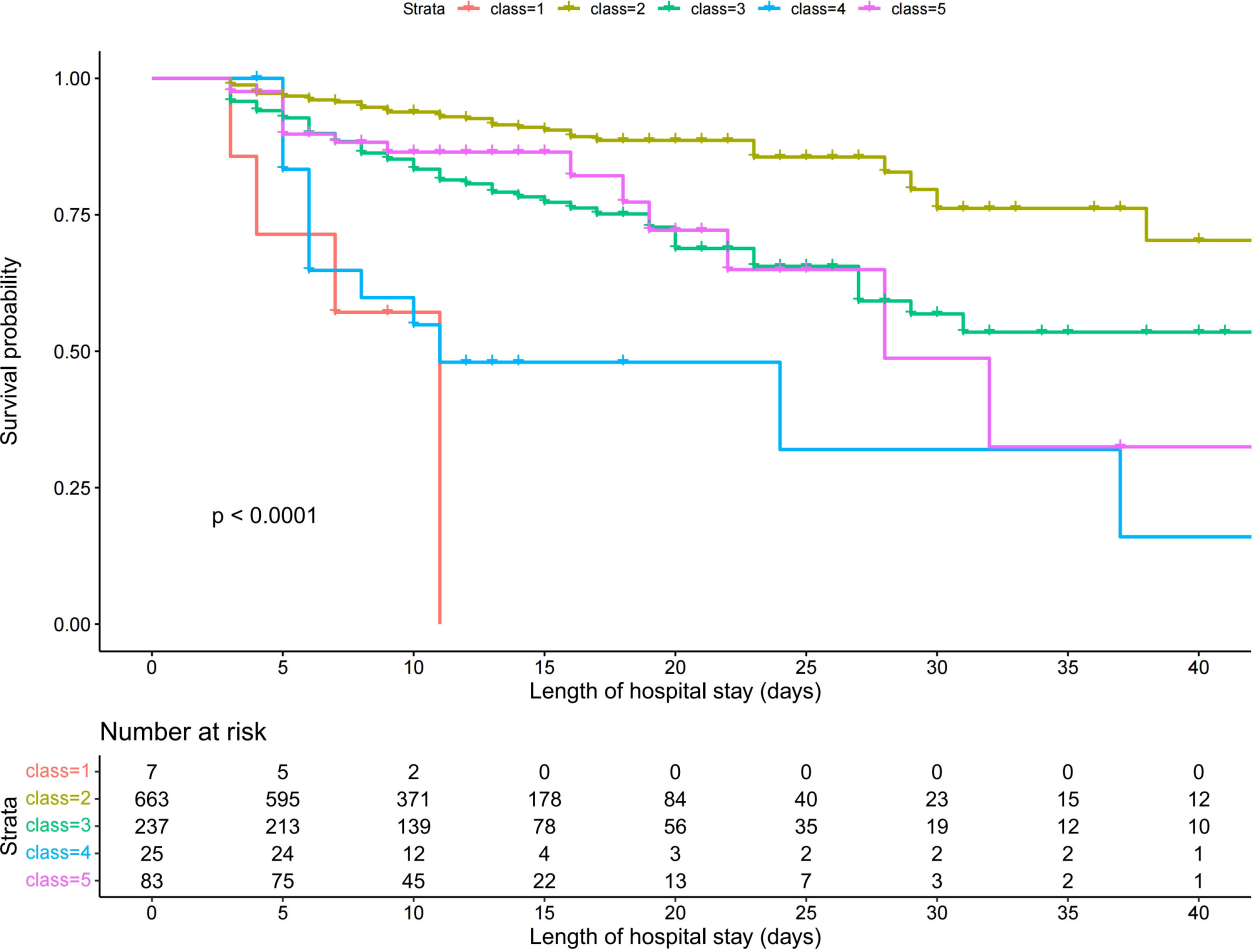
SII logarithmic trajectories of patients with sepsis.

The SII trajectories in Model 5 are shown in **Figure 2**. The specific characteristics of the five subgroups were as follows:

- Class 1 (continuous increase from a low to medium SII level group): accounting for 0.69% of the total sample, characterized by a continuing rapid increase in the SII from a low to medium level;
- Class 2 (stable medium-high SII level group): accounting for 65.32% of the total sample, with SII levels showing a slight decrease but remaining stable at medium-high levels.
- Class 3 (stable decline from high SII level group): accounting for 23.35% of the total sample, the SII in this group gradually decreased from a high level to a medium-high level.
- Class 4 (stable high-level SII group): accounting for 2.46% of the total sample, the SII of this group remained at a high level, with a slight increase but generally stable.
- Class 5 (stable medium-level SII group): accounting for 8.18% of the total sample, the SII of this group remained at a moderate level, with a slight decline but generally stable.

The sex, age, comorbidities, SOFA score, laboratory test results, and outcomes were compared among the five subgroups, and significant differences were found in age and length of ICU stay. There were no statistically significant differences by sex. Except for the lymphocyte count and C-reactive protein level, most of the laboratory test results varied significantly between subgroups,. Among the comorbidities, the prevalence of hypertension, diabetes, coronary atherosclerotic heart disease, cerebrovascular disease, and hematological diseases varied significantly between subgroups, but no statistically significant differences were found among the other comorbidities considered. The SOFA score differed significantly between subgroups. The in-hospital mortality differed significantly between subgroups (**Table 1**), but the length of hospital stay did not differ significantly between subgroups (**Table 2**).

**Table 1:** Baseline characteristics of the sepsis patients with different SII logarithmic trajectory groups at admission.

Variables	Class 1 (n = 7)	Class 2 (n = 663)	Class 3 (n = 237)	Class 4 (n = 25)	Class 5 (n = 83)	*P*
Demographic data						
Age [Median (IQR), year]	63.00 (58.00,64.50)	63.00 (53.00,71.00)	66.00 (55.00,75.00)	69.00 (61.00,77.00)	57.00 (48.00,64.00)	<.001
Male, n(%)	3 (42.86)	344 (51.89)	128 (54.01)	16 (64.00)	46 (55.42)	0.713
SOFA score (points)	6.00 (4.50,11.00)	6.00 (3.00,8.00)	5.00 (3.00,8.00)	5.00 (4.00,7.00)	8.00 (5.00,12.00)	<.001
Comorbidities,n(%)						
Hypertension, n(%)	1 (14.29)	273 (41.18)	106 (44.73)	13 (52.00)	22 (26.51)	0.018
Diabetes, n(%)	2 (28.57)	267 (40.27)	105 (44.30)	10 (40.00)	21 (25.30)	0.046
Coronary atherosclerotic heart disease, n(%)	0 (0.00)	106 (15.99)	47 (19.83)	4 (16.00)	5 (6.02)	0.039
COPD, n(%)	0 (0.00)	20 (3.02)	8 (3.38)	0 (0.00)	0 (0.00)	0.515
Cerebrovascular disease, n(%)	0 (0.00)	94 (14.18)	53 (22.36)	3 (12.00)	8 (9.64)	0.011
Hematologic disease, n(%)	2 (28.57)	7 (1.06)	2 (0.84)	2 (8.00)	2 (2.41)	<0.001
Malignancies, n(%)	1 (14.29)	47 (7.09)	15 (6.33)	3 (12.00)	6 (7.23)	0.795
Liver disease, n(%)	0 (0.00)	21 (3.17)	2 (0.84)	0 (0.00)	4 (4.82)	0.170
Kidney disease, n(%)	2 (28.57)	27 (4.07)	10 (4.22)	2 (8.00)	3 (3.61)	0.083
Immune system diseases, n(%)	0 (0.00)	30 (4.52)	11 (4.64)	2 (8.00)	4 (4.82)	0.816
Laboratory test (Median, IQR)						
White blood cell count (×10^9^/L)	0.41 (0.38,1.64)	12.57 (8.20,18.50)	15.55 (10.39,21.21)	17.27 (13.40,21.63)	9.38 (4.00,16.09)	<0.001
Neutrophil count (×10 ^9^/L)	0.06 (0.01,0.23)	11.26 (6.69,16.38)	13.68 (8.85,19.23)	15.35 (12.52,18.83)	7.49 (2.89,14.40)	<0.001
Lymphocyte count (×10 ^9^/L)	0.33 (0.30,1.06)	0.65 (0.39,1.08)	0.84 (0.41,1.15)	0.68 (0.37,1.04)	0.63 (0.28,1.42)	0.210
Hemoglobin (g/L)	78.00 (72.50,103.00)	122.00 (104.00,138.00)	111.00 (94.00,127.00)	96.00 (77.00,128.00)	118.00 (97.50,139.00)	<0.001
Platelet count (×10 ^9^/L)	13.00 (12.00,72.00)	108.00 (63.50,162.00)	211.00 (150.00,295.00)	251.00 (206.00,359.00)	42.00 (20.50,91.00)	<0.001
C-reactive protein (mg/L)	175.43 (144.99,233.15)	178.90 (100.53,259.94)	195.34 (92.99,274.84)	120.69 (62.39,253.04)	175.97 (93.29,274.97)	0.643
Activated partial thromboplastin time (seconds)	28.90 (25.10,32.15)	30.00 (26.90,33.75)	28.90 (26.00,33.20)	28.10 (26.50,30.30)	36.30 (29.55,45.40)	<0.001
Prothrombin time (seconds)	13.90 (12.55,14.00)	13.40 (12.40,15.00)	13.10 (12.30,14.20)	13.90 (13.00,15.20)	14.10 (12.95,16.15)	0.004
International normalized ratio	1.20 (1.08,1.23)	1.17 (1.08,1.31)	1.15 (1.07,1.25)	1.19 (1.10,1.30)	1.25 (1.11,1.44)	0.022
Fibrinogen (g/L)	5.46 (4.27,6.37)	5.16 (3.74,6.58)	6.33 (4.35,7.84)	4.70 (3.83,6.89)	3.55 (1.96,5.49)	<0.001
Aspartate aminotransferase (U/L)	35.20 (17.95,44.60)	41.90 (25.10,87.55)	30.10 (18.70,58.60)	30.60 (16.40,70.60)	70.10 (42.00,134.65)	<0.001
Alanine aminotransferase (U/L)	21.80 (13.20,32.20)	32.70 (17.80,81.15)	22.90 (14.10,46.50)	21.00 (12.60,41.30)	41.80 (26.80,98.40)	<0.001
Albumin (g/L)	30.20 (27.50,32.95)	30.50 (26.40,34.70)	29.20 (25.20,33.30)	26.40 (24.80,32.30)	28.80 (25.65,32.55)	0.007
Total bilirubin (umol/L)	16.00 (14.85,30.85)	21.10 (13.10,37.20)	16.10 (10.90,29.10)	16.60 (9.30,42.80)	27.00 (17.30,52.65)	<0.001
Direct bilirubin (umol/L)	6.70 (3.70,17.05)	7.80 (4.10,17.35)	5.90 (3.60,11.80)	6.60 (2.50,14.90)	11.30 (5.55,28.85)	<0.001
Creatinine (umol/L)	146.90 (84.10,336.30)	120.80 (81.65,205.50)	104.70 (63.20,191.30)	116.60 (72.60,186.10)	139.30 (75.70,260.35)	0.049
Potassium (mmol/L)	3.54 (3.32,4.33)	3.75 (3.28,4.19)	3.84 (3.34,4.35)	4.12 (3.91,4.50)	3.72 (3.29,4.25)	0.017
Sodium (mmol/L)	133.40 (131.60,136.90)	135.60 (131.70,138.70)	135.00 (130.40,138.90)	135.90 (133.90,140.00)	132.00 (129.10,135.85)	<0.001
Chloride (mmol/L)	105.20 (102.20,105.75)	102.20 (98.15,106.75)	101.20 (97.00,106.10)	103.40 (100.80,108.20)	99.80 (96.55,104.55)	0.014
Calcium (mmol/L)	1.90 (1.85,1.98)	2.03 (1.89,2.15)	2.04 (1.93,2.16)	1.96 (1.86,2.08)	1.93 (1.81,2.10)	<0.001
Troponin I (ng/mL)	0.01 (0.01,0.06)	0.04 (0.01,0.16)	0.03 (0.01,0.13)	0.07 (0.01,0.26)	0.02 (0.01,0.09)	0.049

SOFA score, sequential organ failure assessment score; COPD, chronic obstructive pulmonary disease.

**Table 2:** Clinical outcomes of the study patients with different SII logarithmic trajectory groups.

Variables	Class 1 (n = 7)	Class 2 (n = 663)	Class 3 (n = 237)	Class 4 (n = 25)	Class 5 (n = 83)	P
In-hospital mortality, n(%)	5 (71.43)	56 (8.45)	60 (25.32)	13 (52.00)	16 (19.28)	<0.001
Length of hospital stay, (days)	7.00 (5.50,10.00)	10.00 (7.00,15.00)	11.00 (7.00,19.00)	8.00 (5.00,12.00)	11.00 (6.00,15.00)	0.056
Length of ICU stay, (hours)	161.00 (84.00,162.00)	34.00 (0.00,135.00)	72.00 (0.00,207.00)	119.00 (0.00,240.00)	67.00 (0.00,136.00)	0.005
Continuous renal replacement therapy, n(%)	1 (14.29)	48 (7.24)	27 (11.39)	2 (8.00)	10 (12.05)	0.247
Mechanical ventilation, n(%)	2 (28.57)	89 (13.42)	71 (29.96)	12 (48.00)	21 (25.30)	<0.001

Primary outcomes include overall hospital mortality. Secondary outcomes encompass Length of hospital stay, the length of ICU stay, Continuous renal replacement therapy, Mechanical ventilation.

### 3.2 Relationship between SII trajectory and in-hospital mortality

**Figure 3:**
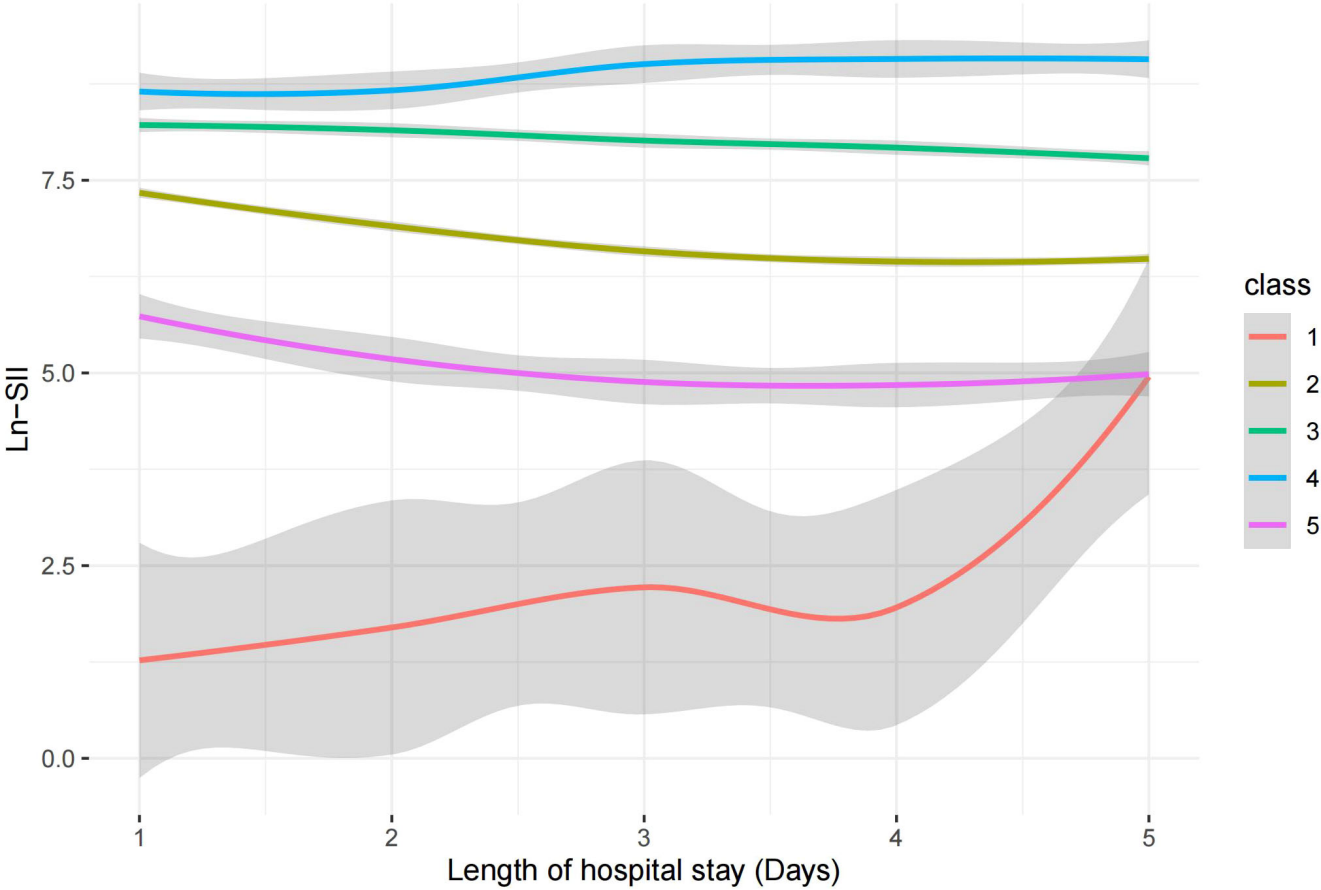
Kaplan–Meier curves for survival, stratified by five classes.

Patients in Class 1 accounted for the highest proportion (71.4%) of in-hospital mortality, whereas patients in Class 2 accounted for the lowest proportion (8.5%) (**Table 2**). Kaplan-Meier survival analysis revealed statistically significant differences in survival between subgroups with different trajectories (**Figure 3**). Class 1 (continuous increase from low to medium SII level) had the highest in-hospital mortality and the fastest decline in survival probability. Class 2 (stable medium-high SII level) had the highest and most stable probability of survival. Class 4 (stable high-level SII) had a survival curve similar to that of Class 1. Class 3 (stable decline from high SII level) and Class 5 (stable medium-level SII) had similar survival curves with higher survival than those of Classes 1 and 4, but lower survival than that of Class 2.

Unadjusted Cox regression analysis confirmed these findings (**Table 3**). Compared with Class 2 as the reference group, the Class 1, Class 3, Class 4, and Class 5 subgroups had a significantly higher risk of in-hospital mortality, with HRs of 15.14 (95% CI: 6.03–38.03) and 2.48 (95% CI: 1.72–3.59), 6.31 (95% CI: 3.43–11.59), and 2.36 (95% CI: 1.35–4.11), respectively. There was no significant difference in mortality risk between Class 3 and Class 5 (HR: 0.95, 95% CI: 0.55–1.65, *P* =0.856) (**Supplementary Table S2**).

**Table 3:** Univariable Cox regression analysis for different SII logarithmic trajectory groups and in-hospital mortality.

Groups	HR	95%CI	P
Class2(reference group)	1		
Class1	15.1424	6.0297 - 38.0270	0.000
Class3	2.4832	1.7170 - 3.5914	0.000
Class4	6.3096	3.4339 - 11.5934	0.000
Class5	2.3586	1.3528 - 4.1122	0.0025

These findings were confirmed using multivariable Cox regression analysis, which included the variables selected through the stepwise regression process (**Supplementary Table S3**). Compared with Class 2 as the reference group, the Class 1, Class 3, Class 4, and Class 5 subgroups had had a significantly higher risk of in-hospital mortality, with HRs of 15.03 (95% CI: 5.79–39.05), 2.13 (95% CI: 1.42–3.19), 5.41 (95% CI: 2.79–10.47), and 2.49 (95% CI: 1.38–4.46), respectively (**Table 4**). After forcibly incorporating septic shock into the multivariate Cox regression model, the association between different trajectory subgroups and increased mortality remained significant. For example, Class 4 exhibited the following: (unadjusted HR: 5.41, P=0.000; adjusted HR: 5.28, p=0.000) (**Supplementary Table S4**).

**Table 4:** Multivariable Cox regression analysis for different SII logarithmic trajectory groups and in-hospital mortality.

Variables	HR	95%CI	P
Class 2	1		Reference
Class1	15.03	5.79-39.05	0.000
Class3	2.13	1.42-3.19	0.000
Class4	5.41	2.79-10.47	0.000
Class5	2.49	1.38-4.46	0.002
Age	1.04	1.02-1.05	0.000
Kidney disease	1.68	0.95-2.97	0.077
Platelet count	1.00	1.00-1.00	0.361
Prothrombin time	1.01	1.00-1.02	0.014
International normalized ratio	1.04	1.02-1.06	0.000
Fibrinogen	0.86	0.79-0.93	0.000

**Figure 4:**
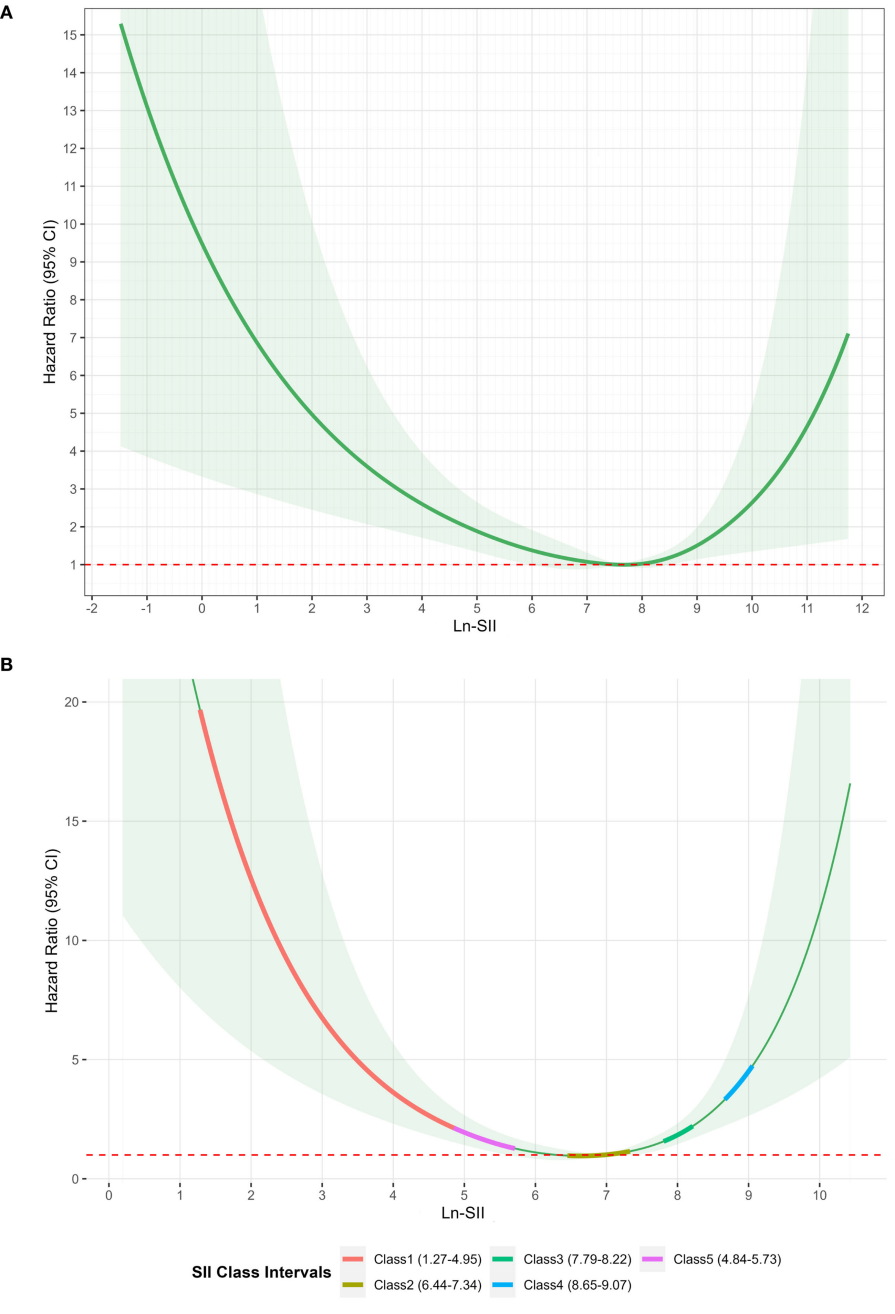
**(A)** Restricted cubic spline of SII logarithmic transformation within 24 hours and in-hospital mortality. **(B)** Restricted cubic spline analysis of the association between 5-day mean log-transformed SII and in-hospital mortality.

Using the mean SII within 24 hours after admission and the mean SII at 5 days after admission as dependent variables, RCS plots were plotted for in-hospital mortality (**Figures 4A, B**). The variation ranges of SII corresponding to the five subgroups were analyzed using a trajectory chart, and the corresponding RCS curve segments of the variation ranges of SII corresponding to different subgroups were marked on the RCS curve with the 5-day mean SII as the dependent variable. The location of the RCS segments corresponding to these subgroups revealed the differences in the risk of in-hospital mortality among the subgroups. The curves for Class 1, Class 3, Class 4, and Class 5 were all above Class 2, consistent with the results of the Kaplan-Meier and Cox regression analyses, showing that, compared with Class 2, Class 1, Class 4, and Class 5 subgroups had a higher risk of in-hospital mortality (**Figure 4B**).

**Figure 5:**
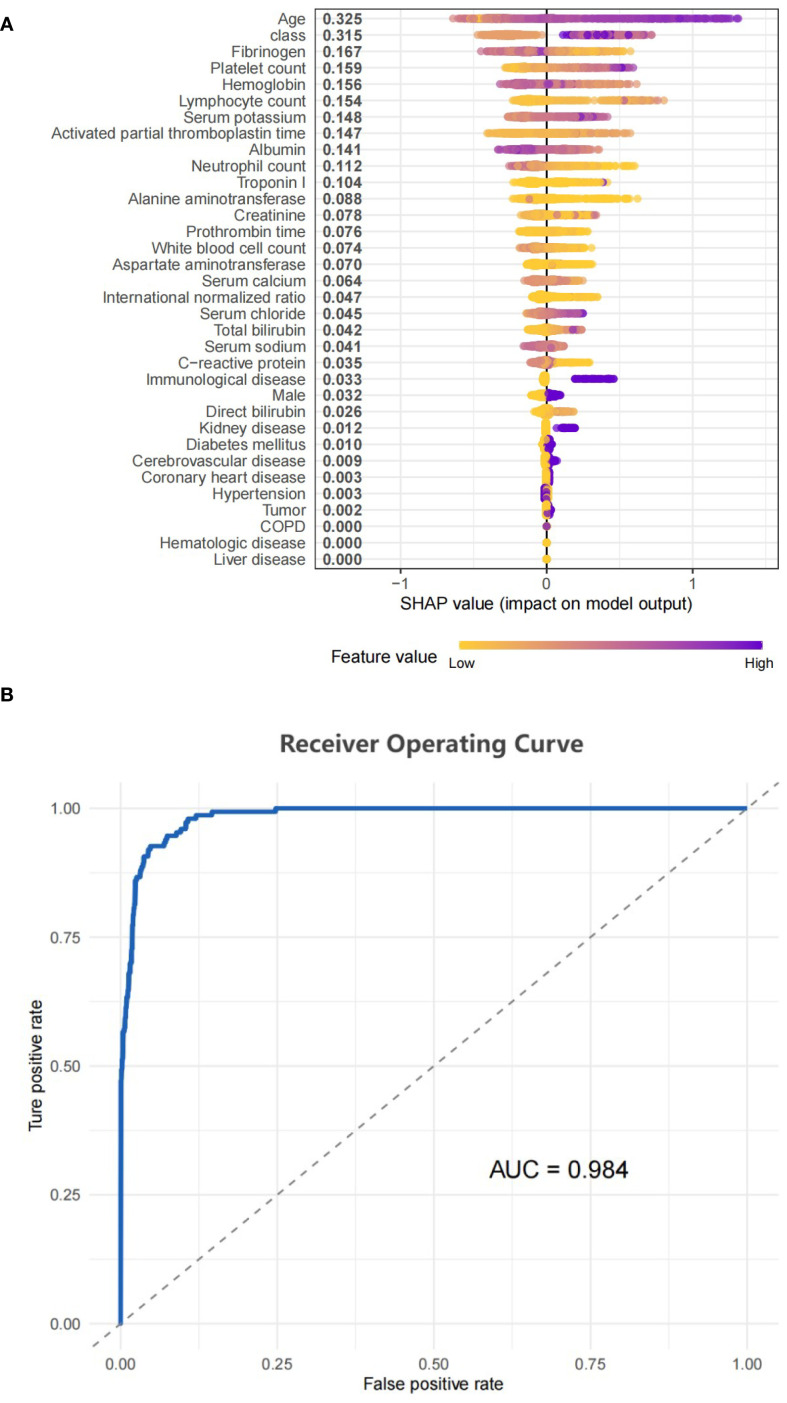
**(A)** SHAP model performance for class. **(B)** Represented the ROC curve for the classifier model.

An XGBoost model was used to analyze the degree of influence of each variable on the risk of mortality in patients with sepsis. This confirmed the independent predictive value of SII trajectory. A SHAP value graph was used to quantify the contribution of each variable to in-hospital mortality (**Figure 5A**). Of the variables considered, age had the highest SHAP value (0.316), followed by Class (0.302), indicating that the different trajectory subgroups were key determinants of the risk of in-hospital mortality. The area under the curve of the XGBoost model was 0.984 (**Figure 5B**), indicating that the model had high sensitivity and specificity in distinguishing the contribution of each variable to the risk of in-hospital mortality.

### 3.3 Nonlinear relationship between SII within 24 hours after admission and in-hospital mortality

The nonlinear association between SII within 24 hours of admission and the risk of in-hospital mortality was statistically significant. According to the RSC plot, the HR showed a U-shaped curve, with patients with low or high SII levels within 24 hours after admission having a higher risk of in-hospital mortality than patients with intermediate SII levels (**Figure 4A**). The HR reached a minimum value at an SII of 7.67. For SII < 7.67, the HR decreased significantly with increasing SII. Conversely, for SII > 7.67, the HR showed a pronounced increase.

## 4 Discussion

This study explored the relationship between SII trajectories and in-hospital mortality in patients with sepsis. The trajectory of SII changes in 1015 patients was analyzed using LCMM, and five distinct SII trajectories with different characteristics were identified. Survival analysis showed that the risk of in-hospital mortality differed among the different SII trajectory subgroups, and was highest in the subgroup with a continuous increase from low to moderate SII levels (Class 1) and the subgroup with stable high SII levels (Class 4). The RCS analysis further revealed a nonlinear relationship between SII within 24 hours of admission and the risk of in-hospital mortality, with both low and high SII levels associated with an increased risk of in-hospital mortality compared with that associated with moderate SII levels. The sample size of Class 1 (n=7) was small. Although supported by statistical model fit indices, this limited sample size renders estimates of baseline characteristics and associated outcomes highly susceptible to random variation. Validation in significantly larger cohorts is warranted before drawing clinical implications.

The SII has shown utility in predicting the prognosis of a variety of diseases and has the advantages of simplicity, low cost, and wide availability. Previous studies have shown that SII is a significant predictor of disease progression and adverse outcomes in the fields of cardiovascular disease, cerebrovascular disease, rheumatology, and immunology (Acar et al., 2021; Weng et al., 2021; Zhang et al., 2021). A study involving 2,543 patients with acute stroke showed that high SII levels were significantly associated with in-hospital complications and poor short-term prognosis (Chu et al., 2020). The 2023 National Health and Nutrition Examination Survey (NHANES) data revealed that an elevated SII was associated with an increased risk of all-cause mortality and cardiovascular mortality in the general population (Xia et al., 2023).

Compared with the findings of previous single-point SII studies, this study revealed differences in the risk of death among different SII trajectory subgroups through the LCMM, with Class 2 (the stable medium-high level group) having the lowest risk. Cox regression analysis confirmed that other subgroups had an in-hospital mortality risk lower than that of Class 2. Similarly, Class 2 had the lowest RCS curve. Over-inhibition of the inflammatory response (illustrated by low SII at the beginning of Class 1) and over-activation of inflammatory response (illustrated by the high SII at the end of Class 4 and the continuous increase of SII at the end of Class 1) are associated with an increased death risk, whereas a moderate inflammatory response (illustrated by Class 2) may help the body to resist infection, reducing mortality risk (Thiery-Antier et al., 2016).

The SII is a composite inflammatory indicator that comprehensively reflects the inflammatory-immune status through the ratio of neutrophil and platelet counts to lymphocyte count (SII = neutrophil × platelet/lymphocyte) (Wang et al., 2016). Compared with a single blood cell or immune cell count, the different subgroups in the SII trajectory model in our study may reflect the potential immune and inflammatory status of sepsis (Islam et al., 2024). The increased and decreased SII reflect different states of the body. An increase in SII indicates a relative increase in platelet and neutrophil counts or a relative decrease in lymphocytes, which is primarily related to enhanced inflammatory response and weakened immune response (Vincent et al., 2002). The release of pro-inflammatory cytokines (such as IL-6, TNF-α, and C-reactive protein) promotes the activation and proliferation of neutrophils and platelets while inhibiting the proliferation of T lymphocytes, inducing lymphocytopenia (Jiang et al., 2022). In sepsis, over-activated neutrophils release inflammatory mediators, which aggravate tissue damage, while platelet activation exacerbates organ failure by promoting vascular endothelial damage and thrombosis. These factors worsen the condition and increase mortality risk in patients (Hurley et al., 2016; Peerapornratana et al., 2019). The subgroup with persistently high SII (Class 4) may represent sustained excessive inflammation, similar to the “cytokine storm phase.” The cytokine storm produced by immune cells somewhat blocks infections; however, it also induces severe tissue damage and organ failure (Chousterman et al., 2017), explaining why this subgroup had a high mortality. This subgroup may need to strengthen the monitoring and prevention of complications caused by excessive inflammatory immune responses and adopt targeted immune regulation strategies to suppress excessive inflammatory responses. Some specific antibodies against inflammatory mediators, such as the IL-1 receptor antagonist (anakinra/rhIL-1ra) found by Knaus et al., prolong the survival time of patients with sepsis (Knaus et al., 1996). Anti-TNF-α monoclonal antibodies effectively prevent shock and organ damage in animal models (Reinhart et al., 2001; Rice et al., 2006). Similarly, anti-IL-3 antibodies reduce neutrophils and inflammatory factors, reduce organ damage, and improve survival rates (Weber et al., 2015). A decrease in SII is primarily related to immune paralysis and bone marrow dysfunction (Claushuis et al., 2016; Li et al., 2019). Persistent inflammatory states trigger bone marrow suppression, manifesting as progressive neutropenia (Jang and Rabb, 2015; White and Ybarra, 2017), thrombocytopenia (Greco et al., 2017), T cell exhaustion, and ultimately developing into an immune paralysis state. This immune paralysis state leads to impaired pathogen clearance and high susceptibility to secondary infections (Torres et al., 2022). For example, in the subgroup with the highest mortality (Class 1), the early stage with a significantly low SII level may be followed by an inflammation rebound owing to secondary infections, resulting in a sustained increase in SII. Such cases may require immune-enhancing therapy in the early stage. For example, granulocyte colony-stimulating factor(G-CSF)/granulocyte-macrophage colony-stimulating factor (GM-CSF)enhances monocyte function and human leukocyte antigen - DR isotype (HLA-DR) expression (Wessendarp et al., 2022), reducing the mortality of premature infants with sepsis; thymosin alfa 1 improves prognosis by regulating immune disorders and reduces the 28-day mortality rate in patients with sepsis (Feng et al., 2016); interferon γ reverses monocyte inactivation during immune paralysis (Payen et al., 2019); IL-7 reverses T cell exhaustion and improves survival rates (Bidar et al., 2022).

The RCS analysis revealed a U-shaped relationship between SII and in-hospital mortality: Ln-SII = 7.67 was the inflection point of risk. At a lower value, the risk of in-hospital mortality decreased with the SII increase; however, at a higher value, the mortality risk increased with the increase in SII. The mortality risk was high when SII was low (immune paralysis) and high (excessive inflammation); it reached its lowest when Ln-SII was controlled at 7.67. A J-shaped relationship has been reported between SII and 28-day mortality in patients with sepsis (Jiang et al., 2023). Low and high SII values are associated with increased short-term mortality risk, and the SII level corresponding to the lowest 28-day mortality risk is 774.46×10^9^/L. We adopted a similar method, and the trends of the impact of SII changes on mortality risk are the same; however, the SII values corresponding to the final inflection point differ. Both studies are retrospective observational studies, and the accurate threshold may require extensive verification in prospective interventional studies.

The condition of patients with sepsis is complex and varies, and patients with septic shock belong to a high-risk group and have a significantly worse prognosis. However, our sensitivity analysis after adjustment for shock status showed that the associations between the trajectory subgroups and mortality remained robust. This suggests that the SII trajectory pattern provides prognostic information beyond the presence of shock. Nevertheless, in the investigation, the inherent pathophysiological and prognostic differences between septic shock and non-shock sepsis were substantial. Therefore, studies specifically examining SII dynamics within the septic shock subgroup are warranted.

This study included patients with sepsis complicated by hematologic diseases because the SII, as a comprehensive indicator of inflammation and immune status, may have prognostic value for such patients (Kochanek et al., 2019). Although patients with hematologic diseases may have aberrant baseline blood cell counts, there were only 15 such patients (1.5%) in this study, constituting a small proportion. Moreover, there were no significant differences in blood cell counts, SII values, or clinical outcomes between the hematologic and non-hematologic disease groups (**Supplementary Table S5**). However, owing to the relatively small sample size, the heterogeneity of the patient population (those with hematologic disease) may not have been fully revealed. Future studies are recommended to be multi-center and large-scale, specifically targeting this special population, to further explore their immune-inflammatory characteristics and prognostic value in sepsis.

A strength of this study is its in-depth analysis of the dynamic changes of SII in patients with sepsis. Most previous studies have focused on the SII measured at a single timepoint and have not considered the dynamic characteristics of sepsis. The LCMM not only identified subgroups of patients with similar longitudinal trajectories, but also enabled the identification of the characteristics of disease progression in different subgroups of patients, providing a targeted basis for personalized treatment. In addition, the use of XGBoost, enabled us to quantify the contribution of each variable to the mortality risk more precisely, improving the interpretability and practicality of the model.

However, this study also has some limitations. First, as a retrospective study, there is potential for selection bias and information bias. Reliance on past medical records may compromise the accuracy and integrity of the data. Secondly, the patients were treated at a single hospital, which may limit the generalizability of the results. In addition, although we controlled for multiple factors that may influence prognosis in the analysis, the possibility of residual confounding cannot be ruled out.

## 5 Conclusion

In this study, the dynamic trajectory of SII in patients with sepsis was analyzed using LCMM, which divided the patients into 5 SII trajectory subgroups. The risk of in-hospital mortality in patients with sepsis differed according to their SII trajectory. The risk of in-hospital mortality increased significantly when SII continued to rise and SII stabilized at a high level, whereas the risk of in-hospital mortality was low when SII stabilized at a medium-high level. RCS analysis revealed a U-shaped relationship between SII within 24 hours of admission and in-hospital mortality, with both low and high SII levels associated with increased risk of in-hospital mortality. The risk of in-hospital mortality was lowest when Ln-SII = 7.67. In this study, the consideration of longitudinal SII trajectories was used to overcome the limitation of traditional single time point measurement. These results provide a dynamic basis for personalized treatment and risk stratification in patients with sepsis.

## Data Availability

The datasets presented in this study can be found in online repositories. The names of the repository/repositories and accession number(s) can be found in the article/**Supplementary Material**.
