# Supplementary material for: Trajectory of the systemic immune-inflammation index and in-hospital mortality in patients with sepsis
Source: Front Cell Infect Microbiol. 2025 Sep 23;15:1616538. doi: 10.3389/fcimb.2025.1616538 (PMC12500555; doi:10.3389/fcimb.2025.1616538)
Supplement: Supplementary file 2 [file Table2.docx]

|  | G | Loglik | Conv | npm | AIC | BIC | SABIC | Entropy | %Class1 | %Class2 | %Class3 | %Class4 | %Class5 | %Class6 |
| --- | --- | --- | --- | --- | --- | --- | --- | --- | --- | --- | --- | --- | --- | --- |
| Model1 | 1 | -6835 | 1 | 6 | 13681.99 | 13711.53 | 13670 | 1 | 100 |  |  |  |  |  |
| Model2 | 2 | -6766.76 | 1 | 10 | 13553.51 | 13602.74 | 13533.53 | 0.1566568 | 3.65 | 96.35 |  |  |  |  |
| Model3 | 3 | -6759.95 | 1 | 14 | 13547.89 | 13616.81 | 13519.91 | 0.3518726 | 4.24 | 91.43 | 4.33 |  |  |  |
| Model4 | 4 | -6748.62 | 1 | 18 | 13533.24 | 13621.84 | 13497.25 | 0.7839688 | 0.69 | 72.71 | 18.23 | 8.37 |  |  |
| Model5 | 5 | -6747.22 | 1 | 22 | 13538.43 | 13646.73 | 13494.45 | 0.9480861 | 0.69 | 65.32 | 23.35 | 2.46 | 8.18 |  |
| Model6 | 6 | -6743.5 | 1 | 26 | 13539 | 13666.99 | 13487.02 | 1.194585 | 0.69 | 57.24 | 2.76 | 22.27 | 11.72 | 5.32 |

Supplementary Table S1. Statistics for choosing the best number of Classes.

AIC, Akaike information criterion; BIC, Bayesian information criteria; SABIC, sample-adjusted information criteria.

Supplementary Table S2. Univariable Cox regression analysis for different SII logarithmic trajectory groups and in-hospital mortality.

| Groups | HR | 95%CI | P |
| --- | --- | --- | --- |
| Class3(reference group) | 1 |  |  |
| Class1 | 6.0980 | 2.4198 - 15.3670 | 0.0001 |
| Class2 | 0.4027 | 0.2784 - 0.5824 | 0.000 |
| Class4 | 2.5409 | 1.3898 - 4.6454 | 0.0025 |
| Class5 | 0.9498 | 0.5454 - 1.6542 | 0.8557 |

Supplementary Table S3. Multivariable Cox regression analysis for different SII logarithmic trajectory groups and in-hospital mortality.

| Variable | HR | CI.Lower | CI.Upper | P |
| --- | --- | --- | --- | --- |
| Age | 1.039 | 1.024 | 1.054 | 0.000 |
| Male | 1.140 | 0.791 | 1.643 | 0.483 |
| Hypertension | 0.751 | 0.510 | 1.105 | 0.147 |
| Diabetes mellitus | 1.135 | 0.786 | 1.640 | 0.499 |
| Coronary heart disease | 1.049 | 0.673 | 1.634 | 0.832 |
| COPD | 0.360 | 0.082 | 1.579 | 0.176 |
| Cerebrovascular disease | 1.144 | 0.735 | 1.783 | 0.551 |
| Hematologic disease | 1.579 | 0.510 | 4.892 | 0.428 |
| Tumor | 1.089 | 0.594 | 1.999 | 0.782 |
| Liver disease | 0.918 | 0.323 | 2.610 | 0.872 |
| Kidney disease | 1.941 | 1.017 | 3.704 | 0.044 |
| Immunological disease | 1.611 | 0.845 | 3.069 | 0.147 |
| White blood cell count | 1.000 | 0.829 | 1.208 | 0.996 |
| Neutrophil count | 0.982 | 0.808 | 1.193 | 0.856 |
| Lymphocyte count | 1.166 | 0.809 | 1.680 | 0.411 |
| Hemoglobin | 0.996 | 0.990 | 1.003 | 0.285 |
| Platelet count | 1.002 | 1.000 | 1.003 | 0.018 |
| C-reactive protein | 1.001 | 0.998 | 1.003 | 0.591 |
| Activated partial thromboplastin time | 1.009 | 0.998 | 1.021 | 0.094 |
| Prothrombin time | 1.013 | 1.003 | 1.024 | 0.014 |
| International normalized | 1.071 | 1.015 | 1.129 | 0.011 |
| Fibrinogen | 0.865 | 0.774 | 0.966 | 0.010 |
| Aspartate aminotransferase | 1.000 | 1.000 | 1.001 | 0.116 |
| Alanine aminotransferase | 0.999 | 0.997 | 1.000 | 0.136 |
| Albumin | 0.994 | 0.960 | 1.030 | 0.745 |
| Total bilirubin | 1.006 | 0.992 | 1.021 | 0.412 |
| Direct bilirubin | 0.998 | 0.976 | 1.021 | 0.878 |
| Creatinine | 1.000 | 0.999 | 1.001 | 0.653 |
| Potassium | 1.160 | 0.947 | 1.421 | 0.151 |
| Sodium | 0.992 | 0.948 | 1.039 | 0.737 |
| Chloride | 1.011 | 0.969 | 1.055 | 0.617 |
| Calcium | 1.460 | 0.768 | 2.777 | 0.248 |
| Troponin I | 1.000 | 0.997 | 1.004 | 0.823 |

Supplementary Table S4. Multivariable Cox regression analysis for different SII logarithmic trajectory groups and in-hospital mortality with septic shock forcibly included in the model.

| Variables | HR | 95%CI | P |
| --- | --- | --- | --- |
| Class 2 | 1 |  | Reference |
| Class1 | 15.32 | 6.08-38.62 | 0.000 |
| Class3 | 2.43 | 1.66-3.56 | 0.000 |
| Class4 | 5.28 | 2.84-9.80 | 0.000 |
| Class5 | 2.14 | 1.20-3.81 | 0.010 |
| Age | 1.04 | 1.02-1.05 | 0.000 |
| Kidney disease | 1.00 | 1.00-1.02 | 0.176 |
| Platelet count | 1.00 | 1.00-1.00 | 0.361 |
| Prothrombin time | 1.01 | 1.00-1.02 | 0.045 |
| International normalized ratio | 1.04 | 1.02-1.06 | 0.000 |
| Fibrinogen | 0.86 | 0.80-0.94 | 0.000 |
| Septic shock | 1.83 | 1.31-2.56 | 0.000 |

Supplementary Table S5. Baseline characteristics of the sepsis patients sepsis with and without hematologic diseases.

| Variables | Sepsis with hematologic diseases (n = 15) | Sepsis without hematologic diseases (n = 1000) | *P* |
| --- | --- | --- | --- |
|  |  |  |  |
| **Blood cell count(×10 ⁹/L)** | | | |
| White blood cell count (×10 ⁹/L) | 9.47(5.035 - 2.315) | 13.225 (8.545 - 18.8425) | 0.388 |
| Neutrophil count (×10 ⁹/L) | 7.96 (1.03 - 21.01) | 11.660 (6.9875 - 16.975) | 0.233 |
| Lymphocyte count (×10 ⁹/L) | 0.63 (0.315 - 1.09) | 0.683 (0.39 - 1.1) | 0.937 |
| Hemoglobin (g/L) | 72.00 (62 - 106) | 119.000 (101 - 137) | 0.000 |
| Platelet count (×10 ⁹/L) | 107.00 (39 - 163) | 122.000 (67.75 - 201) | 0.203 |
| WBC2 (×10 ⁹/L) | 8.07 (5.305 - 20.15) | 12.530 (8.655 - 18.045) | 0.160 |
| NE2 (×10 ⁹/L) | 5.26 (1.55 - 18.835) | 10.780 (6.9975 - 15.87) | 0.122 |
| LYC2 (×10 ⁹/L) | 0.61 (0.385 - 1.065) | 0.840 (0.53 - 1.25) | 0.199 |
| HB2 (×10 ⁹/L) | 63.00 (60.5 - 100) | 109.000 (93 - 127) | 0.000 |
| PLT2 (×10 ⁹/L) | 79.00 (60.5 - 140.5) | 103.000 (55 - 180) | 0.518 |
| WBC3 (×10 ⁹/L) | 10.49 (4.14 - 17.685) | 10.805 (7.545 - 15.675) | 0.837 |
| NE3 (×10 ⁹/L) | 8.63 (0.91 - 15.85) | 8.965 (5.8575 - 13.27) | 0.533 |
| LYC3 (×10 ⁹/L) | 0.73 (0.395 - 1.41) | 0.930 (0.59 - 1.33) | 0.252 |
| HB3 (×10 ⁹/L) | 72.00 (55 - 86.5) | 108.500 (91 - 124) | 0.000 |
| PLT3 (×10 ⁹/L) | 87.00 (61.5 - 139.5) | 105.000 (54 - 177) | 0.572 |
| WBC4 (×10 ⁹/L) | 10.63 (4.49 - 16.455) | 10.240 (7.2975 - 13.8) | 0.936 |
| NE4 (×10 ⁹/L) | 7.64 (2.425 - 14.855) | 8.005 (5.47 - 11.605) | 0.943 |
| LYC4 (×10 ⁹/L) | 0.49 (0.415 - 1.41) | 1.060 (0.68 - 1.51) | 0.055 |
| HB4 (×10 ⁹/L) | 82.00 (67 - 93) | 106.000 (89 - 121) | 0.000 |
| PLT4 (×10 ⁹/L) | 78.00 (57 - 215.5) | 116.000 (64.75 - 194) | 0.416 |
| WBC5 (×10 ⁹/L) | 9.43 (5.545 - 15.105) | 9.410 (6.83 - 12.545) | 0.972 |
| NE5 (×10 ⁹/L) | 7.90 (4.025 - 12.895) | 7.285 (4.8225 - 10.335) | 0.758 |
| LYC5 (×10 ⁹/L) | 0.85 (0.675 - 1.26) | 1.147 (0.77 - 1.55) | 0.216 |
| HB5 (×10 ⁹/L) | 81.00 (66 - 91.5) | 105.500 (89 - 119) | 0.000 |
| PLT5 (×10 ⁹/L) | 104.00 (40 - 228.5) | 138.000 (73.75 - 244) | 0.151 |
| **Systemic immune-inflammation index** | | | |
| SII_day1 | 7.05(4.706 - 8.017) | 7.587 (6.702 - 8.257) | 0.149 |
| SII_day2 | 6.91 (5.408 - 8.160) | 7.129 (6.349 - 7.925) | 0.769 |
| SII_day3 | 6.49 (5.408 - 7.910) | 6.830 (6.097 - 7.635) | 0.575 |
| SII_day4 | 6.69 (5.898 - 8.197) | 6.694 (5.995 - 7.4963) | 0.798 |
| SII_day5 | 6.29 (5.382 - 7.914) | 6.768 (6.022 - 7.430) | 0.450 |
| **Clinical outcomes** | | | |
| In-hospital mortality, n(%) | 4 (26.67) | 146 (14.60) | 0.347 |
| Length of hospital stay, (days) | 8.00 (5.00, 11.50) | 10.00 (7.00, 15.00) | 0.080 |
| Length of ICU stay, (hours) | 72.00 (0.00, 118.50) | 54.50 (0.00, 152.00) | 0.940 |
| Continuous renal replacement therapy, n(%) | 1 (6.67) | 87 (8.70) | 1.000 |
| Mechanical ventilation, n(%) | 2 (13.33) | 193 (19.30) | 0.801 |

WBC, White blood cell count; NE, Neutrophil count; LYC Lymphocyte count; HB, Hemoglobin; PLT, Platelet count.

The numbers following WBC indicate the day on which the WBC measurement was taken. For example, WBC2 represents the WBC value on day 2, and NE3 represents the NE value on day 3.
